# Case Report: Atypical pattern of pathologic response in cutaneous transdifferentiated melanoma with rhabdomyoblastic differentiation following neoadjuvant therapy

**DOI:** 10.3389/fonc.2026.1787950

**Published:** 2026-05-04

**Authors:** Isabela Paulino Serur, Beatriz Mendes Awni Cidale, Ana Zélia Leal Pereira, Gustavo Benfatti Olivato, Giulia Kodja Zanetta, Melissa Lissae Fugimori, Claudia Regina Gomes Cardim Mendes de Oliveira, Maria Fernanda Hunziker, Frederico José Ribeiro Teixeira Júnior, Rodrigo Ramella Munhoz

**Affiliations:** 1 Oncology Department, Sírio-Libanês Hospital, São Paulo, Brazil; 2 Pathology Department Sírio-Libanês Hospital, São Paulo, Brazil

**Keywords:** melanoma, immune checkpoint inhibitors, immunotherapy, neoplasm dedifferentiation, rhabdomyosarcoma, transdifferentiation

## Abstract

Transdifferentiated melanoma is a rare variant of melanoma characterized by phenotypic plasticity, loss of conventional melanocytic markers, and acquisition of features from other tissue lineages, often associated with aggressive biological behavior. Among these, melanoma with rhabdomyosarcomatous differentiation represents an exceptionally rare presentation that poses significant diagnostic and clinical challenges, particularly in the context of immunotherapy. We report the case of a 38-year-old male with a remote history of cutaneous melanoma who presented with an isolated left axillary nodal recurrence. Histopathological examination revealed a biphasic melanoma with a dedifferentiated component showing focal rhabdomyosarcomatous differentiation. The patient was treated with neoadjuvant immunotherapy using ipilimumab and nivolumab. Treatment was discontinued after two cycles due to immune-related hepatitis and aseptic meningitis requiring immunosuppressive therapy. Post-treatment imaging demonstrated a mixed metabolic response. Subsequent axillary lymphadenectomy revealed a complete pathological response of the melanocytic component, while a high-grade sarcomatoid/rhabdomyoblastic component remained viable, exhibiting high proliferative activity and loss of melanocytic markers. Molecular profiling identified NRAS Q61R and TERT promoter mutations, equivocal MYC amplification, and an elevated tumor mutational burden. The patient received adjuvant radiotherapy and remains under close oncologic surveillance. This case illustrates an atypical pattern of pathological response to neoadjuvant immunotherapy, characterized by eradication of the differentiated melanocytic component and persistence of a dedifferentiated, rhabdomyoblastic tumor population. These findings suggest phenotypic plasticity and immune escape as potential mechanisms of resistance, underscoring the need for integrated histopathological and molecular assessment when evaluating pathological response in rare melanoma variants. As neoadjuvant immunotherapy becomes increasingly incorporated into clinical practice, it is crucial to characterize its impact across the diverse presentations of melanoma and to understand the distinct patterns of pathological response.

## 1 Introduction

Transdifferentiated melanoma is a rare variant of melanoma characterized by phenotypic plasticity, with loss of conventional melanocytic markers and acquisition of morphological and immunophenotypic features of other tissue lineages. This phenomenon can lead to misleading histologic and immunohistochemical profiles, particularly in metastatic settings, complicating the differential diagnosis with non-melanocytic malignancies of epithelial, mesenchymal, neurogenic, or hematolymphoid origins (1).

Recent reviews have further highlighted the biological complexity of transdifferentiated melanoma, emphasizing its phenotypic plasticity, diagnostic challenges, and implications for therapeutic response (2).

Among these, melanoma with rhabdomyosarcomatous differentiation is an exceptionally rare presentation, with only a limited number of cases reported in the literature. It is characterized by rhabdomyoblastic transformation, exhibiting both morphological and immunophenotypic features of striated muscle, often accompanied by variable loss of conventional melanocytic markers (3).

Rhabdoid or rhabdomyoblastic tumor cells are typically defined by abundant eosinophilic cytoplasm, eccentric nuclei, and perinuclear aggregates of intermediate filaments (4). Although the molecular mechanisms driving this transdifferentiation remain poorly understood, the expression of mesenchymal and neuroectodermal stem cell markers (such as CD166, CD133, nestin, and MIA) supports the concept of myogenic potential and plasticity of melanoma cells. Immunohistochemically, these tumors often demonstrate immunoreactivity for vimentin, and occasionally for desmin or cytokeratins, while showing reduced or absent expression of melanocytic markers such as HMB-45, Melan-A, and S-100 (5).

In this context, transdifferentiated melanoma refers to the acquisition of a distinct non-melanocytic phenotype, whereas dedifferentiated or undifferentiated melanoma describes tumors that lose melanocytic features without clear lineage-specific differentiation (1, 6). This dedifferentiated phenotype reflects the intrinsic heterogeneity of melanoma and has been associated with tumor progression and resistance to therapies, including immune checkpoint blockade, although these tumors may also exhibit high tumor mutational burden and ultraviolet mutational signatures, suggesting potential sensitivity to immunotherapy (1, 6).

Although prognostic data are limited due to the rarity of this entity, rhabdomyosarcomatous melanomas are generally considered high-grade tumors with aggressive clinical behavior and poor outcomes, often demonstrating resistance to conventional chemotherapy (4, 7). Consequently, there is a growing need to explore alternative therapeutic strategies, especially in advanced disease.

In parallel, the management of resectable stage III and IV melanoma has undergone a paradigm shift with the incorporation of neoadjuvant immunotherapy. Clinical trials such as OpACIN and OpACIN-neo demonstrated the feasibility and optimization of neoadjuvant ipilimumab plus nivolumab (8, 9). The PRADO study further refined this approach by demonstrating that post-surgical management could be tailored according to pathological response, thereby reducing overtreatment and improving patient selection (10). In addition, the SWOG S1801 trial demonstrated improved event-free survival with a neoadjuvant–adjuvant pembrolizumab strategy compared with adjuvant-only treatment, along with higher rates of major pathological response and acceptable safety profiles (11).

More recently, the phase 3 NADINA trial demonstrated that neoadjuvant ipilimumab plus nivolumab significantly improved event-free survival compared with adjuvant nivolumab alone, supporting a response-adapted treatment strategy (12). Updated results with a median follow-up of 25 months confirmed sustained benefit, with improved event-free and distant metastasis-free survival, and identified IFNγ, PD-L1 expression, and tumor mutational burden as predictive biomarkers (12, 13).

These findings have led to the establishment of a new clinical paradigm in which therapeutic decisions are increasingly guided by pathological response to neoadjuvant therapy. However, as neoadjuvant immunotherapy becomes increasingly incorporated into clinical practice, uncommon and biologically distinct pathological response patterns are expected to emerge, particularly in transdifferentiated and dedifferentiated melanoma phenotypes.

In this context, we report the case of a patient with resectable melanoma treated with neoadjuvant ipilimumab and nivolumab, in whom post-treatment pathology demonstrated complete regression of the differentiated melanocytic component with persistence of a high-grade sarcomatoid component.

## 2 Case presentation

This case concerns a previously healthy 38-year-old male with a history of a pigmented lesion on his back, first noted in 2008, which subsequently developed localized bleeding. The patient had no relevant family history of melanoma or other malignancies and no psychosocial factors were identified. In July 2008, he underwent excisional biopsy of a left paramedian lesion, which revealed a diagnosis of cutaneous melanoma with a Breslow thickness of 1.0 mm, Clark level III, and no evidence of ulceration. The patient subsequently underwent wide local excision, which showed no residual tumor as well as sentinel lymph node biopsies of the axillary regions, all of which were negative for metastatic disease. The patient remained under routine surveillance until 2018, when he was formally discharged from oncology follow-up.

In October 2024, the patient noted a bulge in the left axilla, and ultrasound confirmed a suspicious nodular lesion in the left axillary region, measuring 3.8 × 2.7 cm. A biopsy of the left axillary lymph node revealed a malignant spindle and epithelioid cell neoplasm infiltrating fibroadipose tissue. Immunohistochemical analysis showed tumor cell positivity for S-100, CD34, c-Kit (CD117), desmin, smooth muscle actin (SMA), SOX-10, PRAME, and Melan-A. Focal positivity was also observed for HMB-45, trimethylated histone H3 (Lys27), Myogenin, MyoD1, MUM-1, p16, and MTAP. These findings supported the diagnosis of melanoma infiltrating fibroadipose tissue, with a biphasic, dedifferentiated component showing focal rhabdomyoblastic differentiation. Staging with PET-CT and brain MRI showed no evidence of distant metastasis. A detailed timeline of the patient’s clinical course, treatments, and key events is summarized in **Table 1**. No significant diagnostic challenges (e.g., access to diagnostic testing, financial constraints, or delays in evaluation) were encountered during this process.

**Table 1:** Clinical timeline of diagnosis, treatment, and outcomes.

Date	Clinical event	Key findings/interventions
July 2008	Primary melanoma	Excision of cutaneous melanoma; wide local excision and negative sentinel lymph node biopsy
2008–2018	Surveillance	No evidence of disease; discharged from follow-up in 2018
October 2024	Axillary recurrence	Left axillary nodal mass; biopsy consistent with melanoma with focal rhabdomyosarcomatous differentiation; staging negative for distant disease
November 2024	Neoadjuvant therapy	Two cycles of ipilimumab (1 mg/kg) plus nivolumab (3 mg/kg)
November–December 2024	Immune-related toxicity	Immune-related hepatitis and aseptic meningitis requiring immunosuppressive therapy
December 2024	Response assessment	PET-CT showed a mixed metabolic response
January 2025	Surgery	Left axillary lymphadenectomy (levels I-III) and interpectoral lymph node dissection
January 2025	Pathology	Regression of melanocytic component with persistence of a high-grade sarcomatoid tumor showing rhabdomyosarcomatous differentiation
March–April 2025	Adjuvant therapy	Radiotherapy to axillary and regional nodal basins (48 Gy in 20 fractions)
Ongoing	Follow-up	Under close oncologic surveillance

Given these findings, neoadjuvant immunotherapy was initiated with ipilimumab (1 mg/kg) and nivolumab (3 mg/kg). Between November 8 and 28, 2024, the patient received two cycles of treatment. Following the second cycle, he developed immune-related hepatitis and aseptic meningitis, leading to treatment discontinuation and hospitalization for further evaluation and management. He was subsequently discharged on immunosuppressive therapy consisting of methylprednisolone (1 mg/kg) and mycophenolate mofetil, with rapid clinical improvement. Treatment adherence was adequate until discontinuation due to immune-related toxicity, and tolerability was assessed clinically during follow-up.

A follow-up PET-CT performed in December 2024 demonstrated reduced metabolic activity in the left axillary lymphadenopathy (maximum standardized uptake value [SUV] 4.1). However, a focal area in the anterior portion of the lesion showed persistently elevated uptake (SUV 12.3). The lesion had also increased in size, measuring 6.2 cm at its longest axis (previously 5.0 cm), with lobulated contours, heterogeneous density, and possible interspersed necrotic regions (**Figure 1**).

**Figure 1:**
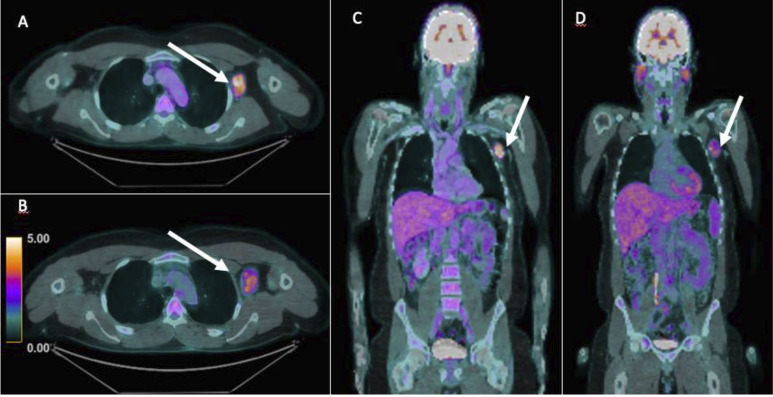
Comparative PET-CT imaging before and after treatment. **(A, C)** Baseline PET-CT (October 2024) **(B, D)** Follow-up PET-CT (December 2024) reveals decreased metabolic activity in most of the left axillary lymphadenopathy (SUV 4.1) and a focal area in the anterior portion of the lesion with persistently elevated uptake (SUV 12.3), consistent with a mixed response to treatment.

On January 14, 2025, he underwent left axillary lymphadenectomy (levels I, II, and III), along with interpectoral lymph node dissection. Pathological examination of the axillary lymphadenectomy specimen revealed fibroadipose tissue free of neoplastic involvement, while the lymphadenectomy specimen showed a high-grade sarcomatoid malignant neoplasm, with no necrosis, a mitotic index of 51 mitoses per 10 high-power fields, no evidence of lymphoid tissue infiltration by the tumor, and no neoplastic involvement in any of the 17 evaluated lymph nodes. Immunohistochemical analysis demonstrated a high proliferative index with Ki-67 staining between 70–80%, supporting the high-grade nature of the tumor. Focal positivity was observed for smooth muscle actin (SMA), desmin, and CD99, indicating mesenchymal and myogenic differentiation. Additionally, focal positivity for Myogenin and MyoD1 provided clear evidence of rhabdomyoblastic differentiation. In contrast, markers typically associated with melanocytic differentiation, including S-100, SOX-10, Melan-A, and HMB-45, and present at baseline, were all negative, indicating loss of the melanocytic phenotype (**Figure 2**).

**Figure 2:**
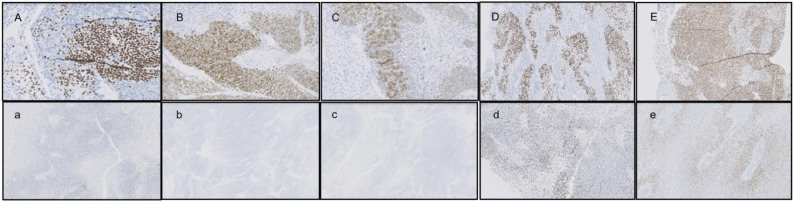
Immunohistochemical staining of melanoma before **(A-E)** and after **(a–e)** treatment. Pre-treatment markers: **(A)** SOX-10, **(B)** HMB-45, **(C)** Melan-A, **(D)** MyoD1, **(E)** PRAME. Post-treatment counterparts **(a-e)** show corresponding markers following therapeutic intervention.

Following multidisciplinary review involving pathology and nuclear medicine specialists, these findings were interpreted as consistent with complete regression of the original differentiated/melanocytic component and persistence of a dedifferentiated, rhabdomyoblastic sarcomatoid component, in keeping with a high-grade sarcomatoid melanoma variant.

Comprehensive molecular profiling was subsequently performed using a next-generation sequencing (NGS) panel targeting gene fusions and clinically relevant genomic alterations of the residual, viable component. No clinically relevant fusions were detected. However, the analysis revealed a pathogenic NRAS Q61R mutation (MAF 43.1%) and a TERT promoter c.-146C>T mutation (MAF 54.9%). An equivocal MYC amplification was also detected (6 copies, chr8q24.21). The tumor mutational burden (TMB) was elevated at 18 mutations per megabase, while microsatellite stability was preserved (MSI-stable).

Therefore, adjuvant radiotherapy was administered to the left axillary region, supraclavicular fossa, and internal thoracic (mammary) chain. Treatment was delivered between March 14 and April 10, 2025, at a total dose of 48 Gy in 20 fractions. The patient tolerated the regimen well and remains under close oncological surveillance. At the most recent imaging assessment (November 24, 2025), there was no evidence of disease.

Timeline summarizing key clinical events from primary melanoma (2008) to axillary recurrence (2024), neoadjuvant ipilimumab plus nivolumab, surgery, adjuvant radiotherapy, and follow-up.

## 3 Methods

### 3.1 Histopathology and immunohistochemistry

The post-treatment axillary lymphadenectomy specimen (levels I–III) was reviewed by dedicated pathologists. Given the clinical history of neoadjuvant immunotherapy, immunohistochemistry was performed on five tumor blocks/samples selected from areas considered most suspicious for residual melanoma. The immunohistochemical panel included epithelial markers (AE1/AE3, EMA, CK7), melanocytic markers (SOX10, S-100, Melan-A, HMB45, PRAME), myogenic markers (smooth muscle actin, desmin, myogenin), and other ancillary markers (CD34, CD99, pan-TRK, H3K27M, INI-1), as well as Ki-67 for assessment of proliferative index. Ki-67 was reported as 70–80%, and INI-1 expression was retained.

### 3.2 Molecular profiling (NGS and fusion testing)

Comprehensive molecular profiling was performed on the viable sarcomatoid tumor component from the lymphadenectomy specimen. The estimated tumor cellularity in the analyzed area was 90%. Next-generation sequencing was carried out using the institutional HSL500 genomic panel (Illumina TruSight™ Oncology 500) and an RNA fusion assay (Archer^®^ FusionPlex^®^ HSL Sarcoma). Sequencing for the TSO500 assay was performed on the Illumina NextSeq 550, and analysis of DNA/RNA data was conducted using DRAGEN TSO500 HT v2.5.2 via Illumina Connected Analytics, with variant prioritization supported by Illumina Connected Annotations. The fusion assay evaluates 67 genes for rearrangements using AMP™-based methodology followed by NGS on Illumina MiSeq, with data analyzed using Archer^®^ Analysis v7.

Tumor mutational burden (TMB) and microsatellite instability (MSI) were reported by the assay; TMB was calculated as eligible coding SNVs divided by the adequately covered genomic territory (mutations/Mb), excluding likely germline variants, and MSI status was derived from analysis of mononucleotide repeat sites using the assay-defined thresholds. Copy-number results were interpreted according to institutional thresholds, with “equivocal amplification” defined as copy number >6 and below the assay threshold for unequivocal amplification.

## 4 Discussion

Transdifferentiated melanoma is a rare and poorly characterized phenomenon, typically arising in the context of advanced disease progression or therapeutic resistance, although these tumors may retain molecular features associated with immunogenicity, including high tumor mutational burden (1).

Among these, melanoma with rhabdomyosarcomatous differentiation is an exceedingly rare and poorly characterized phenomenon. The literature consists predominantly of isolated case reports, and although histopathological features have been increasingly documented, there remains a lack of therapeutic guidance for this distinct variant (3, 14).

Histologically, melanoma with rhabdomyosarcomatous differentiation is characterized by tumor cells exhibiting features of striated muscle differentiation, including abundant eosinophilic cytoplasm, perinuclear filament aggregates, and positivity for myogenic markers such as Myogenin and MyoD1, while often losing expression of conventional melanocytic markers such as SOX-10, S-100, Melan-A, and HMB-45 (3–6).

The immunohistochemical profile of this case is consistent with the findings reported in the literature for melanoma with rhabdomyosarcomatous differentiation, demonstrating complete loss of conventional melanocytic markers (S-100, HMB-45, Melan-A), along with focal expression of Myogenin and MyoD1 (5, 15).

Notably, in our patient, the tumor evolved from a clearly melanocytic phenotype at initial recurrence to a fully dedifferentiated sarcomatoid morphology following neoadjuvant immunotherapy. This phenotypic transformation highlights the known plasticity of melanoma cells, likely stemming from their neural crest origin and retained multilineage potential under therapeutic selective pressure (4, 7).

The concept of melanoma transdifferentiation has gained support from molecular studies demonstrating that these histologic changes often occur without loss of key oncogenic drivers. In our patient, next-generation sequencing confirmed the presence of NRAS Q61R and TERT promoter mutations, consistent with cutaneous melanoma, along with elevated tumor mutational burden (TMB = 18 muts/Mb) and equivocal MYC amplification. These findings parallel those reported by Weigelt et al., who reported a primary cutaneous neoplasm with rhabdomyosarcomatous morphology that closely resembled melanoma both morphologically and molecularly. In their case, next-generation sequencing revealed a mutational profile strongly supportive of melanocytic origin, including NRAS Q61R and TERT promoter mutations, similar to our patient, as well as CDKN2A and TP53 mutations (16). Despite its myogenic phenotype, molecular analysis confirmed a melanoma-like genetic landscape, reinforcing the diagnostic and therapeutic relevance of integrating histopathologic and genomic data in such ambiguous presentations. Ferreira et al. have similarly shown that transdifferentiated melanomas frequently preserve their oncogenic drivers, suggesting that molecular identity is maintained despite dramatic morphological evolution (17, 18).

From a diagnostic perspective, these tumors pose a formidable challenge. Loss of melanocytic markers and acquisition of a myogenic immunophenotype may lead to misclassification as primary sarcomas or other soft tissue tumors (6, 15). PRAME (Preferentially Expressed Antigen in Melanoma) has emerged as a useful diagnostic adjunct in such settings, aiding in confirmation of melanocytic lineage when conventional markers are lost. In a recent study by Choy et al., PRAME expression was retained in 73% of dedifferentiated or transdifferentiated primary cutaneous melanomas, despite complete loss of conventional melanocytic markers. In cases with ambiguous morphology, including those with rhabdomyoblastic features, strong nuclear PRAME staining supported a diagnosis of melanoma (6, 19).

Therapeutic strategies for melanoma with rhabdomyosarcomatous differentiation remain undefined. These tumors are widely considered aggressive and chemoresistant, although sporadic reports have described responsiveness to immunotherapy and radiotherapy (4, 5). Gupta et al. reported a case of metastatic melanoma with rhabdomyosarcomatous differentiation that responded well to combined immunotherapy and palliative radiotherapy. The patient experienced significant tumor reduction and symptom relief, with possible abscopal effect, despite complete loss of melanocytic markers (3). This highlights radiotherapy’s potential role beyond palliation, including immune modulation. Similarly, in our case, adjuvant radiotherapy was chosen after neoadjuvant immunotherapy due to the tumor’s high-grade, dedifferentiated profile and uncertain prognosis.

Neoadjuvant immunotherapy has emerged as a promising approach in resectable stage III melanoma, supported by accumulating evidence from trials such as OpACIN, OpACIN-neo, and PRADO (7–9). The recent phase III NADINA trial further validated this approach, showing that neoadjuvant ipilimumab (1 mg/kg) plus nivolumab (3 mg/kg) administered every three weeks for two cycles, followed by therapeutic lymph node dissection and response-adapted adjuvant therapy, significantly improves outcomes compared to the standard strategy of upfront surgery followed by one year of adjuvant nivolumab. With a median follow-up of 25 months, the neoadjuvant approach achieved 2-year EFS rates of 77.3% versus 55.7% (HR 0.40; 95% CI 0.28–0.57) and 2-year DMFS rates of 82.8% versus 63.9% (HR 0.43; 95% CI 0.29–0.64), and yielded superior depth of pathologic response and early systemic disease control; moreover, IFNγ, PD-L1, and TMB emerged as predictive biomarkers, with biomarker-high subgroups reaching 24-month EFS rates of up to 96–100% in the neoadjuvant arm. Patients achieving a major pathological response (MPR, ≤10% viable tumor) demonstrated particularly favorable outcomes, reinforcing MPR as a clinically meaningful surrogate endpoint (12, 13, 15).

Consensus guidelines, including those from the International Neoadjuvant Melanoma Consortium (INMC), define pathologic complete response (pCR) as the absence of residual invasive tumor in the resected specimen. Other pathologic substages, including near pCR (>0% but ≤10% viable tumor), pPR (>10% to ≤ 50% viable tumor), and no pathologic response (pNR; >50% viable tumor), are increasingly used to guide adjuvant treatment decisions. In general, major pathologic response (≤10% viable tumor) allows for treatment de-escalation, while lesser responses indicate a need for further systemic therapy (20).

Our case raises important questions regarding the interpretation of pathological response in the setting of dedifferentiated or transdifferentiated tumors. While the absence of conventional melanoma cells might suggest a favorable outcome, the persistence of a high-grade sarcomatoid component, known to portend poor prognosis, introduces clinical uncertainty. This scenario underscores the need for refinement in how pathologic response is defined and interpreted, particularly in the setting of rare histological variants.

Emerging evidence suggests that transdifferentiation following immunotherapy may represent a mechanism of immune escape, potentially driven by clonal selection or phenotypic adaptation (7, 21).

Tran et al. described a progression model in which melanoma evolves from conventional histology through an undifferentiated sarcomatoid phase to a rhabdomyosarcomatous phenotype. In their 2019 report, they presented a case of metastatic melanoma harboring a BRAF V600E mutation that initially responded to targeted therapy but relapsed with an undifferentiated sarcoma-like tumor expressing mesenchymal and myogenic markers, ultimately transitioning to a rhabdomyoblastic phenotype with strong desmin and Myogenin positivity. Molecular analysis confirmed clonal relationship across histologic stages, supporting true lineage continuity (22).

Importantly, both the intermediate and final phenotypes could have been misdiagnosed without clinical context and genomic data. Our findings are consistent with this hypothesis and raise the possibility that immune checkpoint blockade, while initially effective, may inadvertently select for resistant dedifferentiated clones.

In conclusion, this case highlights the complexity of melanoma evolution under therapeutic pressure and the limitations of current response-based algorithms. As neoadjuvant strategies become increasingly integrated into standard practice, the field must prepare to address rare and biologically distinct tumor phenotypes. Collaborative registries, centralized pathology review, and integration of molecular diagnostics will be crucial for advancing our understanding and improving outcomes in these challenging cases. Our case contributes to this emerging body of knowledge, providing important insights into the biology, diagnostic considerations, and therapeutic implications of this rare melanoma variant in the immunotherapy era.

This report has several limitations. As a single case, its findings may not be generalizable. In addition, the absence of longitudinal molecular analyses limits the ability to fully characterize the mechanisms underlying phenotypic transformation and therapeutic resistance.

## 5 Patient perspective

The patient reported significant concern upon noticing the axillary mass after a long disease-free interval. He described the immune-related adverse events and hospitalization as physically and emotionally challenging. Following surgery and radiotherapy, he expressed relief with the current clinical status and remains motivated to adhere to close oncologic surveillance.

## Data Availability

This study is a case report, and no datasets were generated or deposited in public repositories. The data supporting the findings are contained within the article and are derived from the patient's clinical records. Due to ethical and privacy considerations, these data are not publicly available. De-identified data may be made available from the corresponding author upon reasonable request and subject to institutional approval.
